# Exploring the Differential Expression and Prognostic Significance of the COL11A1 Gene in Human Colorectal Carcinoma: An Integrated Bioinformatics Approach

**DOI:** 10.3389/fgene.2021.608313

**Published:** 2021-02-01

**Authors:** Ritwik Patra, Nabarun Chandra Das, Suprabhat Mukherjee

**Affiliations:** Integrative Biochemistry & Immunology Laboratory, Department of Animal Science, Kazi Nazrul University, Asansol, India

**Keywords:** bioinformatics, COL11A1 gene, colorectal cancer, mutation, prognosis, survival assay

## Abstract

Colorectal cancer is one of the most common cancers of humans and the second highest in cancer-related death. Genes used as prognostic biomarkers play an imperative role in cancer detection and may direct the development of appropriate therapeutic strategies. Collagen type XI alpha 1 (COL11A1) is a minor fibrillary collagen that has an essential role in the regulation of cell division, differentiation, proliferation, migration, growth, and apoptosis of intestinal and colon cells. The present study seeks to evaluate the significance of the COL11A1 gene in the progression of colorectal cancer in humans across the various parameters using advanced bioinformatics approaches. The application of various databases and servers like ONCOMINE, UALCAN, and GEPIA were accessed for analyzing the differential expression of the COLL11A1 gene and its relative influence over the survival of the transformed subjects. In addition, oncogenomics of COL11A1 gene, mutations associated with this gene and interacting partners of the gene in the context of oncogenesis were studied using COSMIC, cBioPortal, GeneMANIA, and NetworkAnalyst. Our experimental data indicate that the COL11A1 gene is overexpressed in the transformed tissues across the various clinicopathological parameters reduces the probability of survival in both overall and disease-specific survival cases. Mutational studies imply that it can induce perturbations in various signaling pathways viz. RTK-RAS-PI3K, Wnt, TGF-β, and TP53 pathways influencing cancer development. Also, a positive association and correlation amongst the THBS2, COL10A1, COL5A2, and COL1A2 genes were observed, which most likely to contribute to the upregulation of carcinogenesis. Conclusively, this comprehensive study indicates the COL11A1 gene to be a significant contributor in the etiology of colorectal cancer, henceforth this gene can be considered as a prognostic biomarker for the conception of diagnostic and therapeutic strategies against colorectal cancer in the near future.

## Introduction

Colorectal cancer is considered as the third most common cancer in the world and is in the second position for cancer-related death of humans worldwide (Siegel et al., 2017). It is a multi-stage process that gradually develops with the initiation of transformation in normal colon tissue to an adenomatous intermediate by the consequences of mutation, epigenetic changes, DNA damage, uncontrolled growth with gene and chromosomal instability as well as defects leading to invasive adenocarcinoma (Zhang et al., 2011). It is imperative to understand the appropriate mechanism of prognosis, pathogenesis, and genomic alterations associated with colorectal cancer for the development of appropriate therapeutic strategies.

The intestinal extracellular matrix (ECM) is majorly constituted of collagen and is vital for the regulation of cell division, differentiation, proliferation, migration, growth, and apoptosis which signify its cruciality across the development and progression of cancer (Fischer et al., 2001). Collagen type XI alpha 1 (COL11A1) is a minor fibrillary collagen protein, that represents one of the two alpha chains of type XI collagen. Mutations in the COL11A1 gene and/or translational overexpression of COL11A1 protein due to the signaling defects are considered as the essential contributors of carcinogenesis in human colorectal cancer (Raglow and Thomas, 2015). In this context, higher expression of COL11A1 protein has been reported in the cancerous tissue and has been found to be linked with poor progression-free and overall survival across the various types of cancers (Raglow and Thomas, 2015). A microarray-based study reveals that the COL11A1 gene is associated with the disease progression and poor survival in ovarian cancer and regulates cell invasiveness required for tumor formation (Wu et al., 2014). Further studies have also established that COL11A1 gene attributes as a prognostic biomarker for human carcinoma-associated stromal cells and also stimulates cancer progression in lungs, breast, gastrointestinal tract, and pancreas (García-Pravia et al., 2013; Vázquez-Villa et al., 2015; Shen et al., 2016; Li A. et al., 2017; Toss et al., 2019). All these reports collectively suggest that overexpression of COL11A1 in different cancerous tissues results in metastasis and recurrence of several human cancers (García-Pravia et al., 2013; Vázquez-Villa et al., 2015; Shen et al., 2016; Li A. et al., 2017; Toss et al., 2019). COL11A1 is a highly specific biomarker of activated cancer-associated fibroblasts (CAFs) which remains conserved for epithelial cancer irrespective of the site and transformation within the cell undergoing neoplastic transformation, indicating that targeting fibroblast activation could be an effective therapeutic strategy for various cancer (Jia et al., 2016). In an another study, the COL11A1 along with the other two genes viz. THBS2 and INHBA have been found to be overexpressed in colon tissue indicating invasion-facilitated alteration in proteolysis of the extracellular matrix and used for developing high specificity biomarkers sensing cancer invasion and determining response against potential multi-cancer metastasis and therapeutic target (Kim et al., 2010). Particularly for colorectal cancer, previous researchers revealed that the expression of the COL11A1 gene is upregulated up to several folds in the stromal cells of affected colonic mucosa in comparison to the normal tissue (Fischer et al., 2001). Studies on left-sided and right-sided colon cancer, it has been found that COL11A1, TWIST1, insulin-like 5, and chromogranin A were upregulated across the right-sided colon cancer more significantly than that of the left-sided cancer, with a sharp downregulation in 3β-hydroxysteroid dehydrogenase protein (Su et al., 2019). Several experiments on the transformed cells also display significant alteration in a number of cellular signaling pathways, including Wnt, TGF-β, RTK-RAS-PI3K, and TP53 signaling pathways which might be the crucial contributors of the neoplastic transformation (Li et al., 2015; Koveitypour et al., 2019). Although all these various studies imply that the COL11A1 gene is crucial in the progression of various cancer, however, the actual significance across the various clinicopathological factors including cancer-stage, nodal metastasis status, age group, etc., have not been documented comprehensively till date.

The mutations in the COL11A1 gene and resultant impact on the oncogenomic and metabolic pathways are indeed very much essential in understanding the etiology of human colorectal cancer and are yet unclear, thus it provides an area for new research in understanding the actual significance of the COL11A1 gene in the progression of colorectal carcinoma. Regarding this, the application of various bioinformatics tools using the huge dataset of well-established cancer data from different demographic and clinicopathologic patients provides a comprehensive area for further research and development of therapeutic strategies. Considering the background, the objective of the present study is to collectively examine the differential expression, survival, co-expression, correlation, mutations, and protein-protein interaction network that result in the alteration of various pathways related to the COL11A1 gene playing a key role in the transformation of human colon tissue to colorectal cancer using an integrated bioinformatics approach. In addition, our study also aggregates all the available discrete data to identify the significance of the COL11A1 gene as a prognosis biomarker for colorectal cancer which may be useful in designing future research for the conception of appropriate therapeutic strategies.

## Materials and Methods

### Analysis of the Differential Expression of COL11A1 Gene Across Healthy and Transformed Colon Tissues

Differential expression of COL11A1 gene was studied to identify the expression pattern of the COL11A1 gene between tumor and normal tissues across all TCGA (The Cancer Genome Atlas) datasets was performed using TIMER 2.0^1^. It is a comprehensive online resource for systematic analysis of immune infiltrates and gene expression across diverse cancer types (Li T. et al., 2017, Li et al., 2020).

Next, the Oncomine server^2^ was searched for human colorectal cancer and the differential gene analysis section (Cancer vs. Normal Analysis) was selected to retrieve the results. It is a publicly accessible cancer microarray database and web-based data mining platform, containing 715 datasets and 86,733 samples (Rhodes et al., 2004, 2007). The dataset selected for differential expression of mRNA include TCGA colorectal cancer and Kaiser Colon cancer, and recorded within a threshold value of *P*-value- 1E-4, fold change- 2, Gene rank- Top 10 and are shown in Supplementary Table 1.

### Expression Profile and Correlation Analysis

The functional expression of COL11A1 gene in colon carcinoma is analyzed using UALCAN^3^, a public server to analyze the cancer OMICS data (TCGA and MET500), built upon PERL-CGI with high-quality graphics through javascript and CSS to provide graphs and plots depicting gene expression, survival information, epigenetic regulation, and also correlation among gene (Chandrashekar et al., 2017). It is used here to analyze the expression and promoter methylations of the COL11A1 gene in colon adenocarcinoma based on clinicopathological features including sample type, individual cancer stage, patients’ sex and age, histological subtype, nodal metastasis status, and TP53 mutation status and are listed in Supplementary Tables 2,3. The correlation of expression between the COL11A1 with THBS2, COL10A1, COL5A2, and COL1A2 genes for colon adenocarcinoma is performed using the GEPIA^4^ and UCSC Xena^5^ servers (Tang et al., 2017; Goldman et al., 2020).

### Survival Assay of COL11A1 and Its Correlated Genes

The survival analysis for overall survival and disease-free survival is determined by generating Kaplan-Meier (KM) plot using the GEPIA server. It is a web server for analyzing the RNA sequencing expression data of 9,736 tumors and 8,587 normal samples from the TCGA and the GTEx projects (Tang et al., 2017). On the other hand, the KM-plot for disease-specific and overall survival of these genes in the TCGA COAD dataset is performed using the UCSC Xena server.

### Oncogenomics and Mutational Study

cBioPortal^6^ is an online server for exploration, visualization, and analysis of multidimensional cancer genomics data (Cerami et al., 2012). We use it to analyze the impact of the COL11A1 gene in the Colorectal Adenocarcinoma TCGA PanCancer dataset containing 594 samples. It provides a wide range of analysis tab within its server. The oncoprint demonstrates the overview of the COL11A1 gene across the dataset and also generate the heatmap of the correlated gene. Further using the mRNA expression data of the top 25 positively correlated genes, a clustered heatmap is generated using the delimited data on the Clustviz server^7^. The cancer type summary tab provides a detailed overview of the COL11A1 gene across the different subtypes of colorectal cancer i.e., mucinous adenocarcinoma of colon and rectum, colon adenocarcinoma, and rectal adenocarcinoma. It also shows the mutation of the COL11A1 gene for colorectal cancer and the mutational correlation within the associated gene set. The different types of mutations associated with the COL11A1 gene for colorectal cancer were analyzed using COSMIC-“Catalogue of Somatic Mutations in Cancer”^8^ which is the world’s largest source of expert for manually curated somatic mutation information related to human cancers (Tate et al., 2019).

### Analysis for Pathways Associated With the COL11A1 Gene in Colorectal Carcinoma

We have explored PathwayMapper in the cBioPortal server shows the alteration frequencies of selected genes (COL11A1, THBS2, COL10A1, COL5A2, and COL1A1) along with the various pathways overlaid on a TCGA pathway using a white to a red color scale. Furthermore, the top 25 correlated genes belonging to the COL11A1 gene cluster were used to reveal the KEGG (Kyoto Encyclopedia of Genes and Genomes) pathways in Colorectal cancer using DAVID (Database for Annotation, Visualization and Integrated Discovery) available at https://david.ncifcrf.gov/.

### Network and Enrichment Analysis

GeneMANIA^9^ is a web-based platform to determine the association between the gene of interest with other genes using an extensive of functional association data. Herein, this platform was used to analyze the association of the COL11A1 gene with others genes, based on the protein and genetic interactions, pathways, co-expression, co-localization, and protein domain similarity.

After screening, the top 25 significantly correlated gene along with the COL11A1 were used in NetworkAnalyst^10^ for the enrichment analysis including Gene Ontology (GO) enrichment analysis, KEGG and Reactome pathways analysis, and to construct the protein-protein interaction at a generic level using International Molecular Exchange Consortium (IMEx) protein interactions database.

## Results

### Expression of COL11A1 Gene Is Upregulated in Colorectal Cancer

**FIGURE 1:**
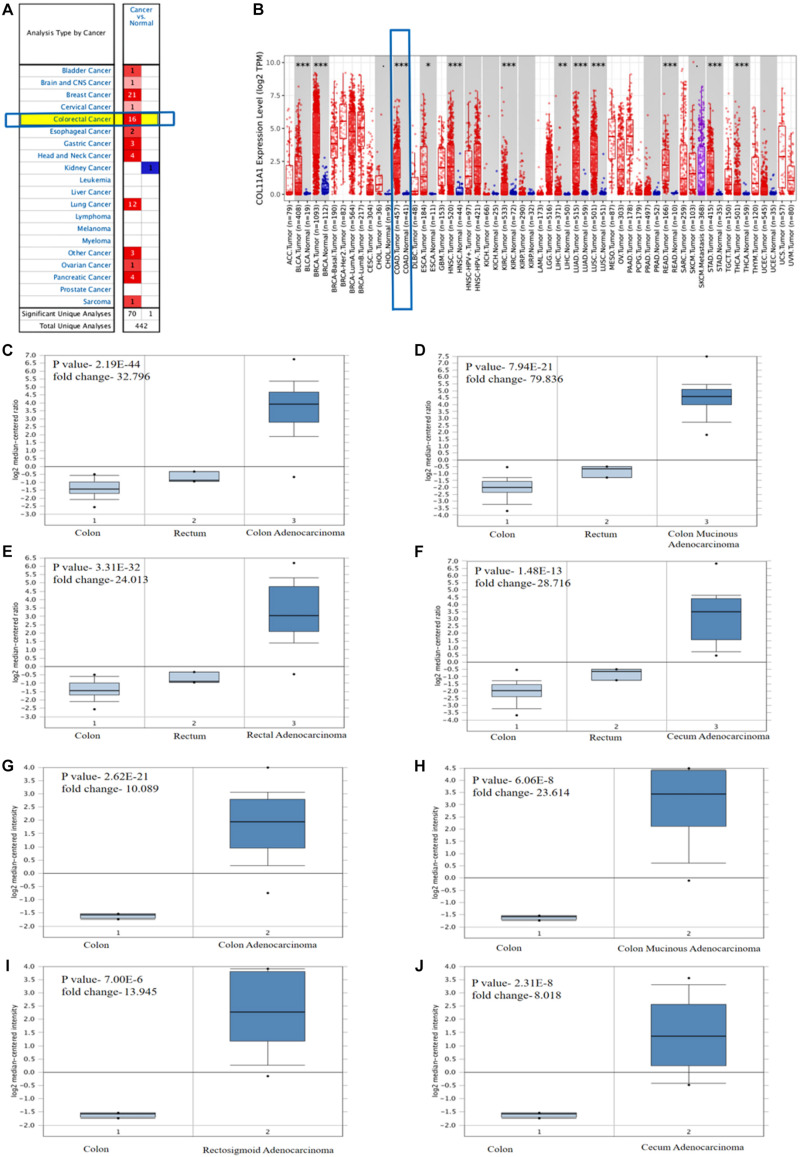
Differential expression of COL11A1 gene **(A)** Expression of COL11A1 mRNA across different cancers where red and blue represent the upregulation and downregulation, respectively. **(B)** Comparative expression of COL11A1 mRNA between colon adenocarcinoma tumor tissue and normal tissue (statistical significance computed by differential analysis, ^∗^*P* < 0.05; ^∗∗^*P* < 0.01; ^∗∗∗^*P* < 0.001). **(C–F)** Box plot comparison of COL11A1 expression for TCGA colorectal cancer dataset in panel **(C)**. Colon adenocarcinoma, **(D)** Colon Mucinous Adenocarcinoma, **(E)** Rectal Adenocarcinoma, **(F)** Cecum Adenocarcinoma. **(G–J)** Box plot comparison of COL11A1 expression for Kaiser colon cancer dataset in panel **(G)**. Colon adenocarcinoma, **(H)** Colon Mucinous Adenocarcinoma, **(I)** Rectosigmoid Adenocarcinoma, **(J)** Cecum Adenocarcinoma.

The role of the COL11A1 gene in colorectal cancer is significantly upregulated in colorectal cancer (Figure 1A). The TIMER analysis reveals that the comparison of the COL11A1 gene across various cancer types including colon cancer and displays that it is significantly upregulated for colon adenocarcinoma (Figure 1B). Further analyses of the mRNA expression profiles of the COL11A1 gene in normal and transformed tissue in ONCOMINE server reveal significant upregulation of COL11A1 mRNA in both the subtypes of cancer datasets i.e., TCGA colorectal cancer and Kaiser colon cancer (Figures 1C–J and Supplementary Table 1). It includes colon adenocarcinoma (*p*-value- 2.19E-44, fold change- 32.796), colon mucinous adenocarcinoma (*p*-value-7.94E-21, fold change- 79.836), rectal adenocarcinoma (*p*-value-3.31E-32, fold change- 24.013), and cecum adenocarcinoma (*p*-value-1.48E-13, fold change- 28.716) for TCGA colorectal cancer (Figures 1C–F), and is somehow greater than that of the Kaiser Colon cancer dataset (Figures 1G–J). All these data collectively indicate that human colorectal cancer samples display significantly higher expression of COL11A1 mRNA in comparison to normal colon and rectum tissues, indicating COL11A1 could have a crucial role in the neoplastic transformation of colorectal cancer.

### Transcriptional Expression and Epigenetic Regulation of COL11A1 Across Various Clinicopathological Parameters

**FIGURE 2:**
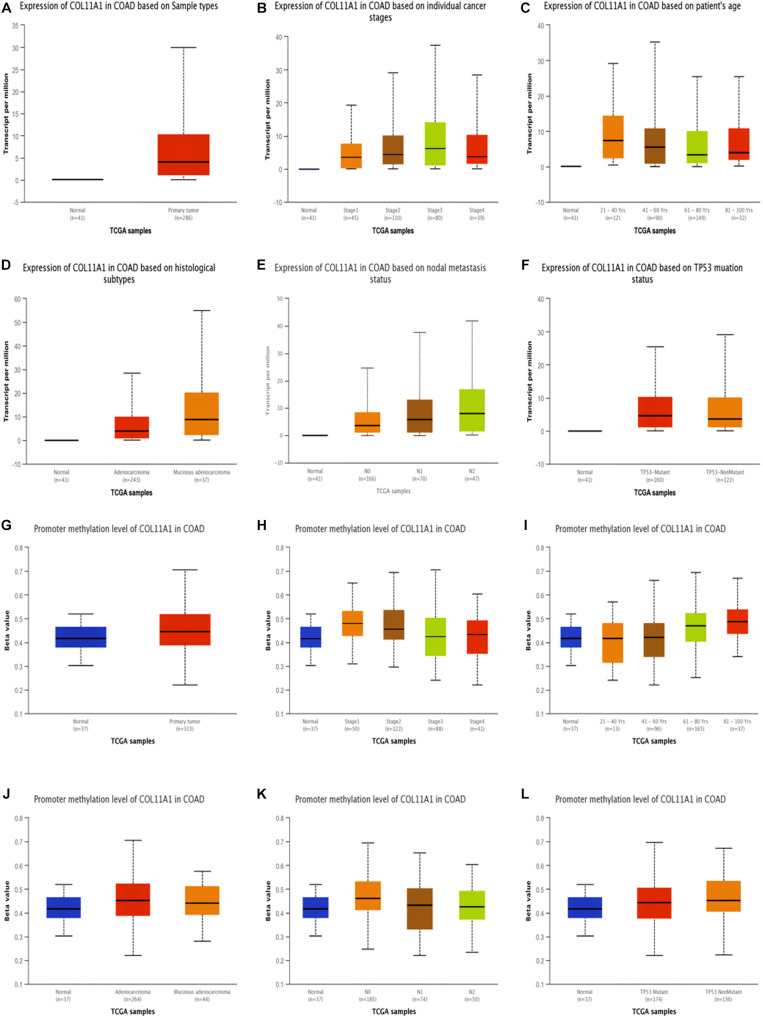
Expression and promoter methylation of the COL11A1 gene in colon adenocarcinoma for different clinicopathological parameters. **(A–F)** Box-plot showing relative expression of COL11A1 mRNA in panel **(A)**. cancer tissues and normal tissues, **(B)** individual cancer stage, **(C)** patient’s age, **(D)** histological subtypes, **(E)** nodal metastasis status, **(F)** TP53 mutation status. **(G–L)** Box-plot showing promoter methylation of COL11A1 mRNA in, **(G)** cancer tissues and normal tissues, **(H)** individual cancer stage, **(I)** patient’s age, **(J)** histological subtypes, **(K)** nodal metastasis status, **(L)** TP53 mutation status.

The expression of COL11A1 in colon adenocarcinoma was analyzed based on the different clinicopathological parameters like sample type, individual cancer stage, patient’s sex and age, histological subtype, nodal metastasis status, and TP53 mutation status using the UALCAN server (Figure 2 and Supplementary Table 2). The results support the inference depicted in the earlier section by demonstrating that COL11A1 expression is higher in the colorectal cancer tissue at different clinical stages than in normal tissue (Figure 2A). It tends to increase the expression of COL11A1 at advanced stages of cancer (Stage 3 > Stage 2 > Stage 1) (Figure 2B) and decrease along with the increase in the age group of patients (Figure 2C). It was also found that the expression of the COL11A1 gene increases along with the nodal metastasis status (N2 > N1 > N0) (Figure 2E).

DNA methylation is relatively associated with the development of cancer within the human body (Greenberg and Bourc’his, 2019). From our data, it was evident that the promoter methylation of the COL11A1 gene is overexpressed in the colon cancer tissue than that of the normal tissue, and is negatively regulated for all other clinicopathological parameters (Figures 2G–L and Supplementary Table 3). It is reflected that along with the development of cancer stages and nodal metastasis status, the expression of promoter methylation decreases in the tissues (Stage 1 > Stage 2 > Stage 3; N0 > N1 > N2) (Figures 2H,K). These results indicate that the promoter methylation is negatively associated with the expression of COL11A1 mRNA, and the hypermethylation of the promoter of COL11A1 may inhibit COL11A1 in upgrading cancer development.

### Survival Assay of the COL11A1 Gene in Colorectal Cancer

**FIGURE 3:**
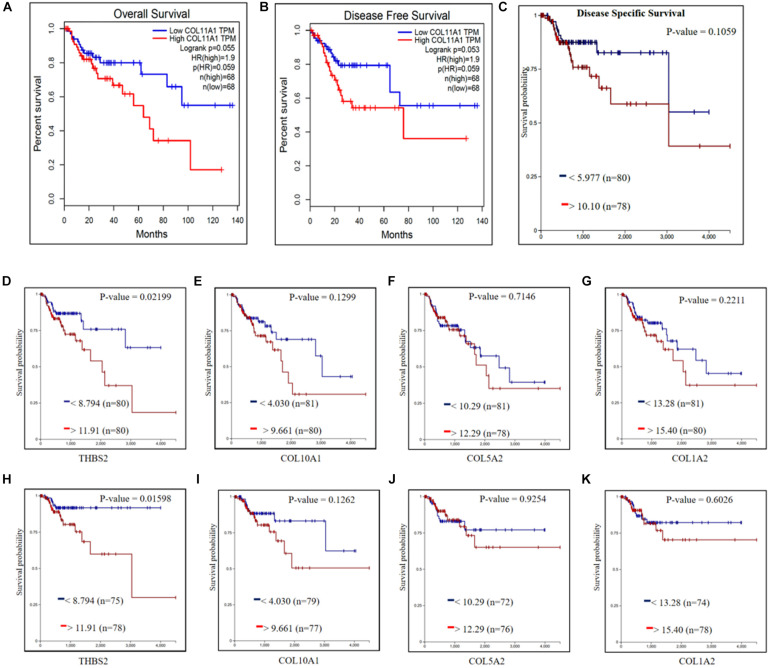
KM-plot for survival assay of COL11A1 and other associated genes. **(A–C)** Effect of COL11A1 expression on **(A)** overall survival, **(B)** Disease-free, **(C)** Disease-specific survival. **(D–G)** Overall survival concerning gene expression of **(D)** THBS2, **(E)** COL10A1, **(F)** COL5A2, **(G)** COL1A2. **(H–K)** Disease-specific survival for the gene expression of **(H)** THBS2, **(I)** COL10A1, **(J)** COL5A2, **(K)** COL1A2. (Red and blue indicate the higher and lower expression of the gene, respectively).

Survival analysis is one of the key components in analyzing the influence of any cancer-associated gene (Clark et al., 2003). In this study, the survival assay of the COL11A1 gene is explained by the KM-plots which show a reciprocal correlation between the expression of COL11A1 and overall survival (log-rank p- 0.055) or disease-free survival (log-rank p-0.053), which signifies the COL11A1 gene as a poor prognostic indicator for colorectal cancer (Figures 3A,B). Also, the disease-specific survival plot of COL11A1, obtained from the UCSC XENA server indicates that higher expression leads to lower survival probability (*p*-value- 0.1059) (Figure 3C). Therefore, low COL11A1 expression in colorectal cancer patients is correlated with prolonged survival, but high COL11A1 expression in colorectal cancer is associated with poor survival.

The survival assay of the correlated genes shows similar significance to that of the COL11A1 gene in the colon adenocarcinoma dataset. The KM-plot obtained for overall survival at higher expression of THBS2 (*p*-value- 0.021), COL10A1 (*p*-value- 0.129), COL5A2 (*p*-value- 0.714), and COL1A2 (*p*-value- 0.221) is related with lower survival probability (Figures 3D–G). Similarly, the disease-specific survival is also decrease with the increase in expression of THBS2 (*p*-value- 0.015), COL10A1 (*p*-value- 0.126), COL5A2 (*p*-value- 0.925), and COL1A2 (*p*-value- 0.602) (Figures 3H–K).

### Co-expression and Correlation Amongst the Other Genes Associated With COL11A1 in Colorectal Cancer

**FIGURE 4:**
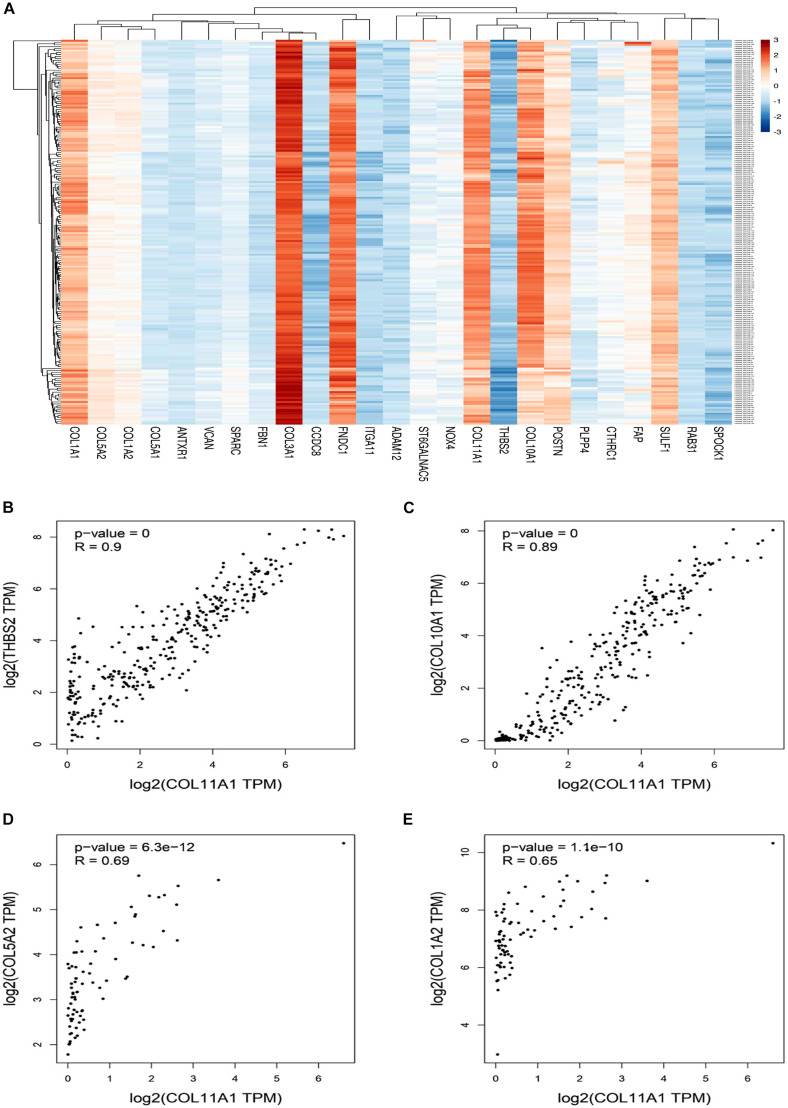
Coexpression and correlation of genes functionally associated with COL11A1. **(A)** Clustered heatmap of the top 25 correlated genes (Scaling in –3/3 with mean-centered to 0). **(B–E)** Graphical representation of Pearson’s correlation test of COL11A1 gene with, **(B)** THBS2, **(C)** COL10A1, **(D)** COL5A2, **(E)** COL1A2.

The top 25 positively co-expressed genes were analyzed via cBioPortal, containing the Spearman’s correlation coefficient, *p*-value from two-sided *t*-test, and also *q*-value derived from the Benjamini-Hochberg FDR correction procedure (Supplementary Table 4). Further mRNA expression data was used for generating a clustered heatmap showing expression between +3/−3 with mean-centered to 0 (Figure 4A). From these above two analyses, it is found that the co-expression of THBS2, COL10A1, COL5A2, and COL1A2 is most likely to be positively correlated with the COL11A1 gene in colorectal cancer (Table 1). To further validate the co-expression, another heatmap was generated using UCSC XENA server to correlate the gene expression of the associated genes with respect to the COL11A1 gene, represented as a histogram with the z score transformation (Supplementary Figure 1). Moreover, correlation graph was obtained using the Pearson’s correlation coefficient amongst COL11A1 gene with THBS2 (*R*-value- 0.90), COL10A1 (*R*-value- 0.89), COL5A2 (*R*-value- 0.69) and COL1A2 (*R*-value- 0.65) (Figures 4B–E). Collectively all these results reveal that the COL11A1 gene has a positive association and correlation with THBS2, COL10A1, COL5A2, and COL1A2 to upregulate the gene expression to induce the development of colorectal cancer.

**TABLE 1:** The correlation among the genes associated with COL11A1 gene in colorectal cancer.

Correlated Gene	Spearman’s correlation	Pearson’s correlation
THBS2	0.922	0.90
COL10A1	0.913	0.89
COL5A2	0.909	0.69
COL1A2	0.903	0.65

### Genomic Alteration and Mutation Associated With COL11A1 Gene in Colorectal Cancer

**FIGURE 5:**
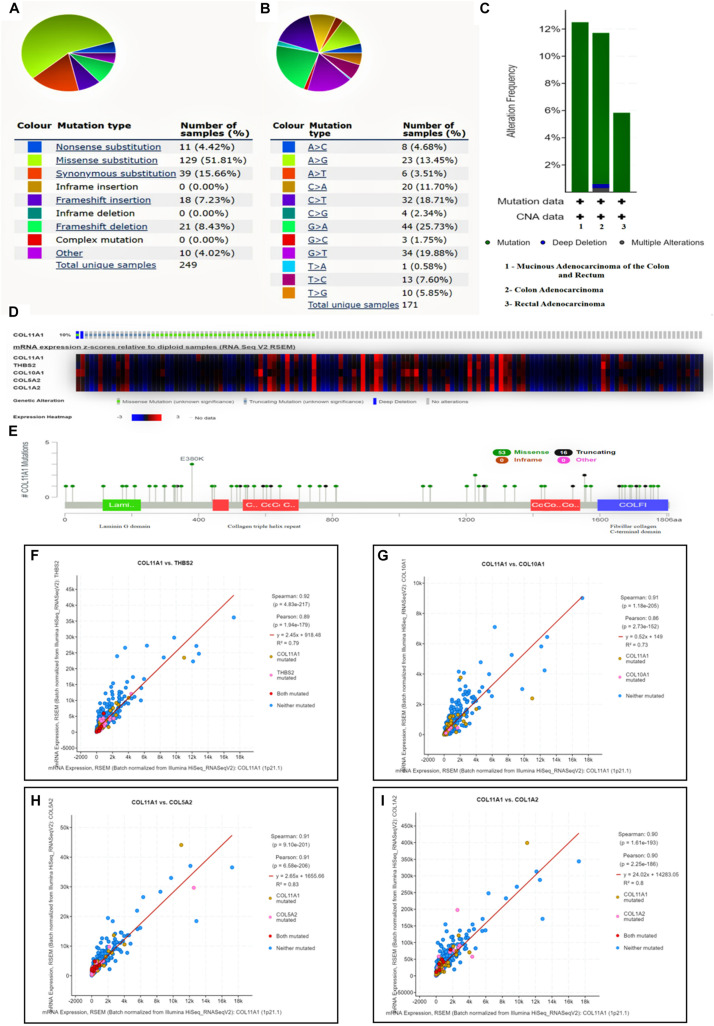
Mutational analysis of COL11A1 gene. **(A)** Summary of various types of mutations associated with COL11A1 gene. **(B)** Bar-graph depicting various types of substitutional mutation occurring within the gene. **(C)** Mutation along the subtype of cancer including mucinous adenocarcinoma of colon and rectum (>12%), colon adenocarcinoma (<12%), and rectal adenocarcinoma (∼6%). **(D)** Oncoprint showing mutational rate of COL11A1 gene and the heatmap for mRNA expression of associated genes. **(E)** Genomic information of COL11A1 mutation. **(F–I)** Graphical representation of correlation between COL11A1 gene showing mutation, Pearson correlation coefficient, Spearman correlation coefficient and regression line with, **(F)** THBS2, **(G)** COL11A1, **(H)** COL5A2, **(I)** COL1A2.

The COL11A1 gene mutation was analyzed on COSMIC database comprising more than 2406 samples of colorectal cancer out of which 249 were recorded for mutations, among them the missense substitution is highest with 51.81% followed by synonymous substitution (15.66%), frameshift mutation (15.66%), nonsense substitution (4.42%) and other types (4.02%) (Figure 5A). The breakdown of various substitution mutation is shown in Figure 5B, representing the highest type of G > A (25.73%) and lowest showing T > A (0.58%). To determine and analyze the frequency and type of mutation, cBioPortal server was used where the cancer type summary indicates the mutation along with the various subtypes of colorectal cancer showing mucinous adenocarcinoma of colon and rectum (>12%), colon adenocarcinoma (<12%), and rectal adenocarcinoma (∼6%) (Figure 5C). The Oncoprint and Mutation tab shows that the COL11A1 gene is altered in 10% of the total 526 patients in TCGA colorectal cancer dataset along with the heatmap for the associated genes (Figure 5D). Additionally, a mutational study for the correlation among the COL11A1 gene with THBS2, COL10A1, COL5A2, and COL1A2 (Figures 5F–I) showing a significant coefficient value for both Spearman and Pearson Correlation test and the regression line. It is observed that the mutation of COL11A1 is much more expressive for COL1A2 > COL5A2 > THBS2 > COL10A1.

### Gene Network and Pathways Alteration

**FIGURE 6:**
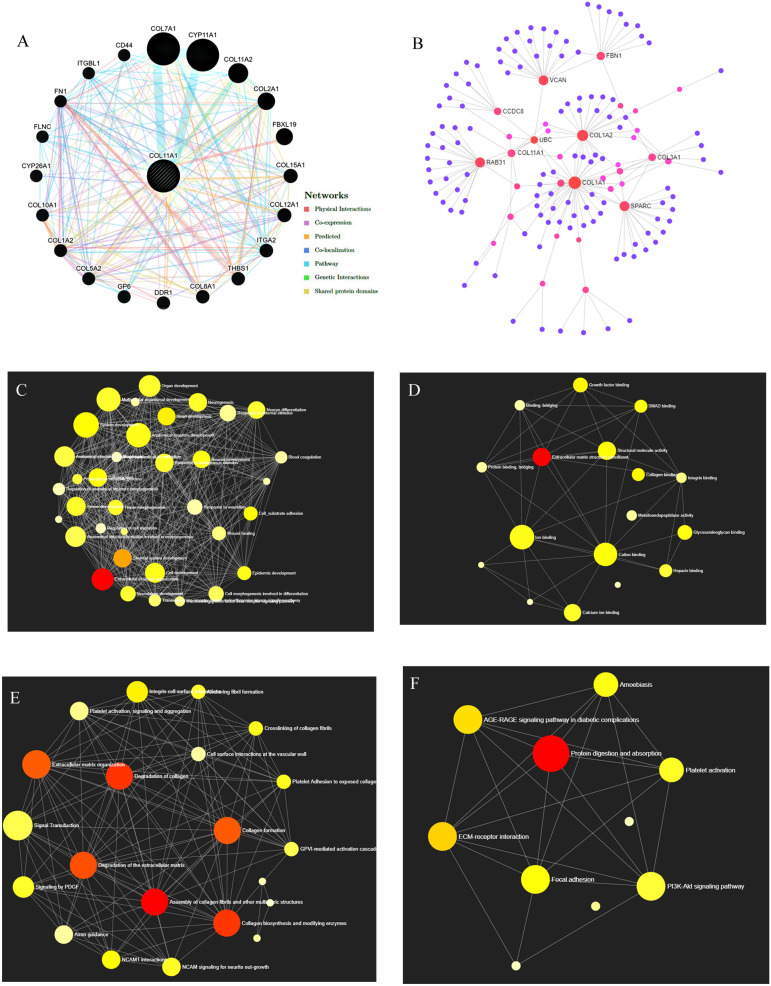
Gene Network Analysis. **(A)** COL11A1 gene with its neighboring genes showing physical interactions (67.64%), coexpression (13.50%), predicted (6.35%), co-localization (6.17%), pathways (4.35%), genetic interaction (1.40%), and shared protein domains (0.59%) **(B)** Protein-protein interaction network based on IMEx protein interactions database. **(C–F)** Network enrichment analysis for **(C)** GO: Biological process, **(D)** GO: Molecular function, **(E)** Reactome pathways, and **(F)** KEGG pathways.

GeneMANIA server provides a complete network of COL11A1 gene with its neighboring gene of interaction in colorectal cancer displaying the physical interactions (67.64%), coexpression (13.50%), predicted (6.35%), co-localization (6.17%), pathways (4.35%), genetic interaction (1.40%), and shared protein domains (0.59%) (Figure 6A). The Gene Ontology (GO) enrichment analysis was performed on NetworkAnalyst to obtain the network of GO: biological pathway (Figure 6C), and molecular function (Figure 6D) showing the significance of the genes in extracellular structure organization, collagen fibril organization, protein complex subunit organization, collagen metabolic process, cell migration, etc., and are listed in Supplementary Table 5. It was further used to generate the network for Reactome (Figure 6E) and KEGG pathway (Figure 6F) analysis. Moreover, the protein-protein interaction (PPI) network was constructed based on the International Molecular Exchange Consortium (IMEx) protein interactions database using NetworkAnalyst represented the crucial protein and helps to further establish the genes promoting in colorectal cancer prognosis and development. As shown in the PPI network (Figure 6B), the degree of a node is the number of connections among the node, and betweenness is the smallest path amongst nodes showing RAB31 (Degree:19, Betweeness:2401.3), COL1A1 (Degree:36, Betweeness:5357.51), COL1A2 (Degree:26, Betweeness:2955.04), COL3A1 (Degree:9, Betweeness:657.29), COL11A1 (Degree:7, Betweeness:537.23), and VCAN (Degree:18, Betweeness:2701.11) as the important proteins of the network.

**FIGURE 7:**
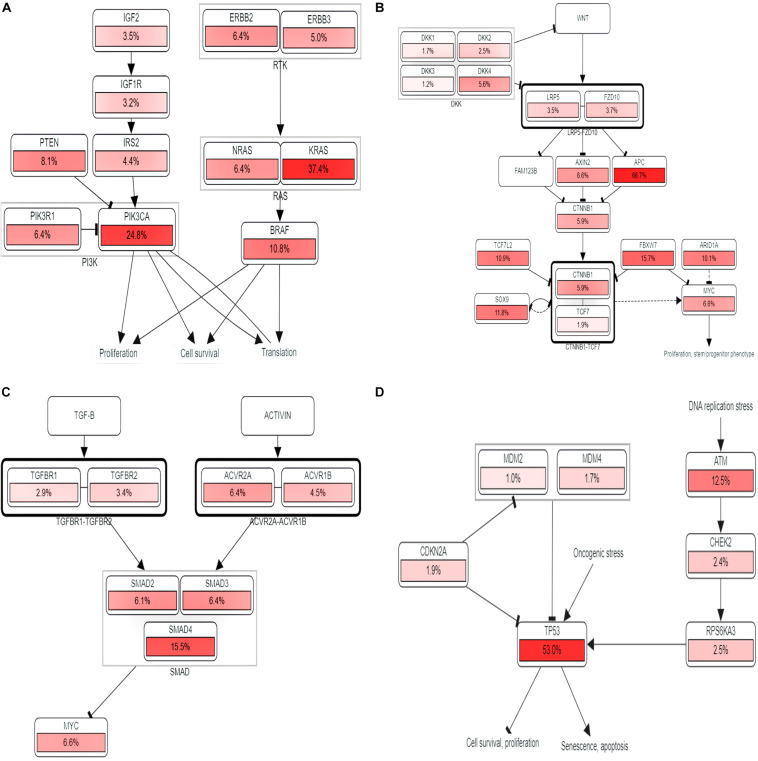
Pathway analysis. **(A–D)** Impact of COL11A1 and associated gene in regulating alteration frequency of **(A)** RTK-RAS-PI3K signaling pathway, **(B)** Wnt signaling pathway, **(C)** TGF-β signaling pathway, **(D)** TP53 pathway.

The KEGG pathways established from the DAVID analysis indicate the intervention of COL11A1 and associated genes in the ECM-receptor interaction (Supplementary Figure 2A), Protein digestion and absorption (Supplementary Figure 2B), Focal-adhesion (Supplementary Figure 2C), and PI3K-Akt signaling pathway (Supplementary Figure 2D), and are listed in Table 2. The PathwayMapper tab in cBioPortal servers shows the alteration frequency of COL11A1, THBS2, COL10A1, COL5A2, and COL1A2 over the various pathways on the colorectal cancer dataset using a white to red color scale where the more frequently altering gene shows greater intensity of the red color (Figures 7A–D). COL11A1 associated alteration mainly induces changes of PTEN (8.1%), PIK3CA (24.8%), KRAS (37.4%), and BRAF (10.8%) for regulation of RTK-RAS-PI3K signaling pathway (Figure 7A); APC (66.7%) in regulation of Wnt signaling pathway (Figure 7B); SMAD4 (15.5%) for TGF-β signaling pathway (Figure 7C); and ATM (12.5%) and TP53 (53.0%) in alteration of TP53 pathway (Figure 7D) to proliferate the cancer development.

**TABLE 2:** KEGG pathways analysis using the DAVID server for top 25 correlated genes of COL11A1 in Colorectal cancer.

Pathways	Gene count	Percentage	Fold enrichment	*p*-value	*q*-value
ECM-receptor interaction	8	30.8	52.7	1.3E-11	2.1E-10
Protein digestion and absorption	7	26.9	45.6	1.6E-9	1.3E-8
Focal-adhesion	8	30.8	22.3	5.8E-9	3.1E-8
PI3K-Akt signaling pathway	8	30.8	13.3	2.1E-7	8.3E-7

## Discussion

In this modern era, the change in lifestyle, food habits, consumption of carcinogens, and several altered environmental factors are collectively considered as the major concerns of colorectal cancer and related deaths. The functional association amongst the various genetic and epigenetic processes are known to play a remarkable role in the initiation and progression of colorectal cancer (Pancione et al., 2012). In particular, overexpression and differentiation of ECM molecules, including collagen in the intestine, are considered as the key determinants of the proliferation and development of colorectal cancer (Fischer et al., 2001). The COL11A1 gene is a minor fibrillary collagen and plays an essential role in the fibrillogenesis and skeletal morphogenesis by controlling the lateral growth, and interfibrillar spacing of collagen II fibrils (Brown et al., 2011). Hitherto, studies available in the literatures and databases provide discrete evidences on the regulation of COL11A1 gene expression in the onset of various types of carcinomas (Vázquez-Villa et al., 2015; Toss et al., 2019). Our present study is a maiden attempt to provide a comprehensive knowledge of the various clinical relevance of the COL11A1 gene in the expression profile, methylation, survivability, and mutation in association with the colorectal cancer.

The mRNA expression profile of COL11A1 gene obtained from TCGA dataset of colorectal cancer from the various servers like ONCOMINE, UALCAN, and GEPIA collectively discloses significant upregulations at transcriptional level in cancer tissue than the normal colon tissue across various cancer subtypes including colon adenocarcinoma, colon mucinous adenocarcinoma, rectal adenocarcinoma, and cecum adenocarcinoma (Figures 1C–J); and even in the various clinicopathological parameters including patients’ age, cancer stage, nodal metastasis status, and TP53 mutation (Figures 2A–F). Epigenetic changes in the gene are known to be the leading causes of neoplastic transformation, and regarding this, our result on the promoter methylation of the COL11A1 gene across various parameters indicates negative relation with the expression profile in a way suggesting the hypermethylation of the COL11A1 gene may regulate the of development cancer (Figures 2G–L). The KM-plots obtained for the overall survival (Figure 3A) and disease-free survival (Figure 3B) show poor prognosis of colorectal cancer i.e., the higher expression of the COL11A1 gene signifies poor survivability. The coexpression and correlation of the top 25 positively correlated genes with the COL11A1 gene are depicted on the heatmap (Figure 4A). Herein, we have found that THBS2, COL10A1, COL5A2, and COL1A2 are the most significant gene having the highest positive correlation (Supplementary Figure 1 and Figures 4B–E). Further, upon the survival assays of THBS2, COL10A1, COL5A2, and COL1A2 genes it has been found a similar pattern of lower survival probability on overexpression (Figures 3D–K). Collectively all these experimental data clearly reveal that the COL11A1 gene along with its associated THBS2, COL10A1, COL5A2, and COL1A2 might serve as a prognostic biomarker for colorectal cancer.

The genomic alteration and mutation are the major inducers for the initiation and development of several cancers (Loeb et al., 2008). In our study, it has been observed that up to 12% mutation that relates to the COL11A1 gene contributes to the development of colorectal cancer with the highest alteration in mucinous adenocarcinoma of colon and rectum (Figure 5C). Further analysis from the COSMIC server illustrates that substitution mutation is the most prevalent mutation that constitutes the highest frequency of G > A types of changes (Figures 5A,B). In addition, the prevalence of THBS2, COL10A1, COL5A2, and COL1A2 enhances the frequency of alteration and depicting a positive correlation with COL11A1 mutation (Figures 5F–I). A functional network of the interaction among the neighboring genes of the COL11A1 in colorectal cancer displays physical interactions, co-expression, predicted co-localization, pathways, genetic interaction, and shared protein domains (Figure 6A). Collectively, we can postulate that the COL11A1 gene interacts with the neighboring mediators to induce the downregulation of various biological signaling pathways. Enrichment network created through NetworkAnalyst shows GO enrichment of various biological and molecular pathways where the genes significantly associated with extracellular structure organization, collagen fibril organization, protein complex subunit organization, collagen metabolic process, and cell migration (Figures 6C,D and Supplementary Table 5). The PPI networks indicate the RAB31, COL1A1, COL1A2, COL3A1, COL11A1, and VCAN as the most important protein network that likely to be connected and show betweenness among themselves to significantly promote the prognosis of colorectal cancer (Figure 6B).

The KEGG pathways established from DAVID analysis reveals that it shows the highest intimacy with the ECM-receptor interaction and PI3K-Akt signaling pathway (Supplementary Figure 2). Moreover, our study through the PathwayMapper tab of the cBioPortal website indicates the frequency of alteration of the various signaling cascades of RTK-RAS-PI3K, Wnt, TGF-β, and TP53 pathways that consequently leads to colorectal cancer. The RTK-RAS-PI3K signaling axis is important in regulating the cell growth and survival (Xu et al., 2020). Perturbation in these signaling cascades is known to contribute in the induction as well as in the development of cancer. The mutation of KRAS is found to be higher in colorectal cancer and thought to enhance the malignancy character of the transformed cells (Zenonos and Kyprianou, 2013). The alteration of the PI3K pathway mainly including the RTK upstream regulator of PI3K, catalytic subunit PIK3CA, PTEN negative regulator, and the downstream regulator of PI3K lead to the surge of cancer development (Yuan and Cantley, 2008). Herein, our study reveals the impact of COL11A1 gene product in the alteration of PTEN, PIK3CA, KRAS, and BRAF which might downregulate the RTK-RAS-PI3K signaling pathways to induce cancer development (Figure 7A). On the other hand, the Wnt signaling pathway is associated with the regulation of various developmental and physiological processes including cell division, specification, proliferation, and even maintenance of tissues and abnormal signaling leading to colorectal cancer (Clevers, 2006). The mutation of APC leads to overactivation of Wnt signaling pathways resulting in 80% of colorectal cancer prognosis (Koveitypour et al., 2019). The influence of COL11A1 and its associated gene triggers alteration of APC for around 66.7% that resulted in the overactivation of Wnt signaling pathways leading to cancer development (Figure 7B). TGF-β signaling pathway plays a vital role in tissue maintenance and is associated with inflammation and carcinogenesis by restraining the cell growth, differentiation, and apoptosis (Koveitypour et al., 2019). Mutation of TGF-β receptor type 2 (TGFBR2) leads to the microsatellite instability causing colorectal cancer, and also the loss of function of SMAD4 in the TGF-β signaling pathway promotes the tumor progression and poor survival in colorectal cancer (Itatani et al., 2019). The alteration of the TGF-β signaling pathway by the COL11A1 gene indicates that the SMAD4 alteration frequency of 15.5% might drive the formation of cancer (Figure 7C). TP53 pathway is the regulator of the cell cycle, DNA replication, apoptosis, and response to a wide range of stresses and safeguards maintenance of genomic integrity and acts as a tumor suppressor gene (Aubrey et al., 2016). The mutation of TP53 leads to colorectal cancer elevating the invasiveness, metastasis, and poor survival (Li et al., 2015). The association of the COL11A1 gene with its correlated gene from our study influences the alteration of TP53 by 53.0% disrupting the pathway results in uncontrolled cell proliferation and metastasis (Figure 7D).

**FIGURE 8:**
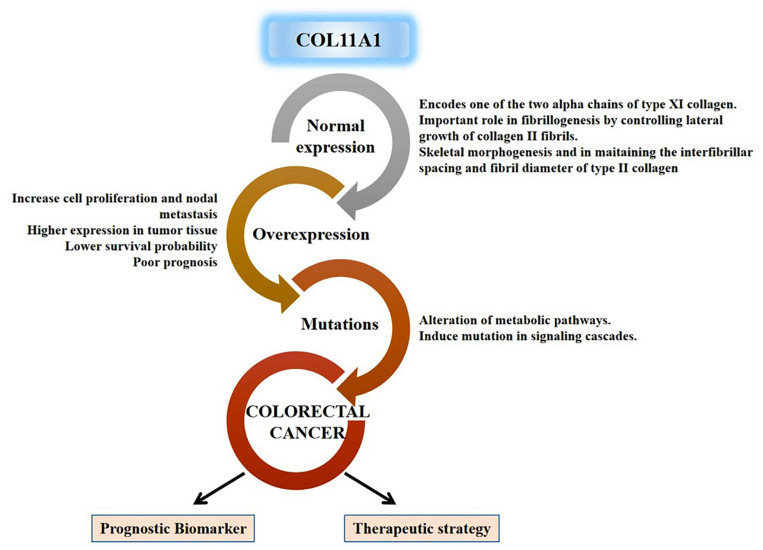
Schematic representation for functional relevance of COL11A1 gene in the oncogenesis of colorectal cancer and its candidature as a prognostic biomarker and therapeutic target.

All these discrete pieces of evidences from our experimental results designate the significance of the COL11A1 gene along with its highly correlated genes (THBS2, COL10A1, COL5A2, and COL1A2) in the progression of colorectal cancer across the various parameters using a wide range of data available in the cancer databases globally. Taken together, this study comprehensively enlightens the validation of the COL11A1 gene in the initiation, progression, and development of colorectal cancer using the bioinformatic approach, and the overall mechanism is schematized in Figure 8.

## Conclusion

Our study provides several important pieces of evidences on the significance of the COL11A1 gene in the prognosis of human colorectal cancer. The overexpression of COL11A1 is positively upregulated in the cancer tissue across the various clinicopathological conditions, while negatively regulated in the case of promoter methylation indicating that the hypermethylation can induce the inhibition of cancer development. The survival assay signifies poor prognosis in both overall and disease-free survival. Our *in silico* study reveals that an abundance of COL11A1 mRNA could induce the transcriptional upregulation of THBS2, COL10A1, COL5A2, and COL1A2 genes cooperatively, to promote the neoplasia. The dysregulation in the expression of COL11A1 and mutations alters various critical regulatory pathways to influence the oncogenesis of colorectal cancer in humans. Therefore, our experimental data firmly claims the candidature of the COL11A1 gene as a potential biomarker for the prognosis of colorectal cancer and opens new areas of research for the diagnosis and development of appropriate therapeutic strategies. However, further *in vitro* and *in vivo* experimental validations are required to determine the efficacy of the COL11A1 gene in the prognosis of colorectal cancer and the development of a therapeutic strategy. Regarding this, we are in order to work on the cancer cell-lines and the murine model of colorectal cancer for validating the present study and developing efficacious therapeutic strategy by targeting COL11A1 gene.

## Data Availability Statement

The original contributions presented in the study are included in the article/Supplementary Material, further inquiries can be directed to the corresponding author.

## Author Contributions

RP performed all the experiments, analyzed the data, and wrote the manuscript. NCD reviewed the data and manuscript. SM analyzed the data, edited the manuscript, and supervised the study. All authors contributed to the article and approved the submitted version.

## Conflict of Interest

The authors declare that the research was conducted in the absence of any commercial or financial relationships that could be construed as a potential conflict of interest.

## Footnotes

1 http://timer.cistrome.org/

2 https://www.oncomineorg/

3 http://ualcan.path.uab.edu/

4 http://gepia.cancer-pku.cn/

5 https://xena.ucsc.edu/

6 https://www.cbioportal.org

7 https://biit.cs.ut.ee/clustvis/

8 https://cancer.sanger.ac.uk/cosmic

9 https://genemania.org

10 https://www.networkanalyst.ca/
