# Supplementary material for: Exploring the Differential Expression and Prognostic Significance of the COL11A1 Gene in Human Colorectal Carcinoma: An Integrated Bioinformatics Approach
Source: Front Genet. 2021 Feb 1;12:608313. doi: 10.3389/fgene.2021.608313 (PMC7882494; doi:10.3389/fgene.2021.608313)
Supplement: Supplementary file 1 [file Data_Sheet_1.PDF]

Exploring the differential expression and prognostic significance of the COL11A1 gene in human colorectal carcinoma: an integrated bioinformatics approach

Ritwik Patra[1], Manojit Bhattacharya[2]*, Nabarun Chandra Das[1], Suprabhat Mukherjee[1]*

Affiliations:

[1] Integrative Biochemistry & Immunology Laboratory, Department of Animal Science, Kazi Nazrul University, Asansol-713340, West Bengal, India

[2] Department of Zoology, Fakir Mohan University, Vyasa Vihar, Balasore- 756020, Odisha, India

**** Correspondence.***

Dr. Suprabhat Mukherjee,
Department of Animal Science,
Kazi Nazrul University,
Asansol- 713340; West Bengal, India
E. mail: *suprabhat.mukherjee@knu.ac.in*

Dr. Manojit Bhattacharya,
Department of Zoology,
Fakir Mohan University,
Vyasa Vihar,
Balasore- 756020, Odisha, India

Email: *mbhattacharya09@gmail.com*

SUPPLEMENTARY MATERIALS

Suppl.Table 1- Expression of COL11A1 among various dataset of colorectal cancer subtype and normal individuals using the Oncomine database at threshold of P-value- 1E-4, fold change- 2, Gene rank- Top 10.

Dataset	Subtype of Colorectal Cancer	P-value	Fold change	T test
TCGA Colorectal cancer	Colon Adenocarcinoma	2.19E-44	32.796	27.871
	Colon Mucinous Adenocarcinoma	7.94E-21	79.836	19.840
	Rectal Adenocarcinoma	3.31E-32	24.013	19.357
	Cecum Adenocarcinoma	1.48E-13	28.716	12.368
Kaiser Colon cancer	Colon Adenocarcinoma	2.62E-21	10.089	17.346
	Colon Mucinous Adenocarcinoma	6.06E-8	23.614	10.870
	Cecum Adenocarcinoma	2.31E-8	8.018	9.436
	Rectosigmoid Adenocarcinoma	7.00E-6	13.945	8.364

Suppl.Table 2- The COL11A1 mRNA expression for Colon adenocarcinoma based on different clinicopalthoogical parameters using UALCAN.

Variables	Different stages	N	Comparisons	Statistical significance
Sample types	Normal	41	Normal vs. primary tumor	1.62458935193399E-12
	Primary tumor	286		
Individual cancer stages	Stage 1	45	Normal vs. stage 1	5.170200E-04
	Stage 2	110	Normal vs. stage 2	8.30830004794336E-09
	Stage 3	80	Normal vs. stage 3	5.43420000109762E-08
	Stage 4	39	Normal vs. stage 4	1.538910E-03
Patients age	21-40 yrs	12	Normal vs. 21-40 yrs	5.498700E-03
	41-60 yrs	90	Normal vs. 41-60 yrs	6.62479999746779E-08
	61-80 yrs	149	Normal vs. 61-80 yrs	1.22389987033955E-10
	81-100 yrs	32	Normal vs. 81-100 yrs	1.51568999999796E-05
Histological subtype	Adenocarcinoma	243	Normal-vs-Adenocarcinoma	1.11022302462516E-16
	Mucinous adenocarcinoma	37	Normal-vs-Mucinous-adenocarcinoma	1.968210E-04
Nodal metastasis status	N0-No regional lymph node metastasis	166	Normal-vs-N0	3.00499625183193E-11
	N1- Metastases in 1 to 3 axillary lymph nodes	70	Normal-vs-N1	6.03890000006047E-07
	N2-Metastases in 4 to 9 axillary lymph nodes	47	Normal-vs-N2	6.3018999999942E-05
TP53 mutation status	TP53 mutant	160	Normal-vs-TP53-Mutant	2.18125517648105E-12
	TP53 non-mutant	122	Normal-vs-TP53-NonMutant	5.55159995752064E-09

Suppl.Table 3- The COL11A1 promoter methylation for Colon adenocarcinoma based on different clinicopathological parameters using UALCAN.

**Variables**	**Different stages**	**N**	**Comparisons**	**Statistical significance**
Sample types	Normal	37	Normal vs. primary tumor	5.476900E-03
	Primary tumor	313		
Individual cancer stages	Stage 1	50	Normal vs. stage 1	3.197100E-03
	Stage 2	122	Normal vs. stage 2	6.26710000000141E-05
	Stage 3	88	Normal vs. stage 3	4.995600E-01
	Stage 4	41	Normal vs. stage 4	7.005000E-01
Patients age	21-40 yrs	13	Normal vs. 21-40 yrs	6.607600E-01
	41-60 yrs	96	Normal vs. 41-60 yrs	9.255400E-01
	61-80 yrs	165	Normal vs. 61-80 yrs	1.990770E-04
	81-100 yrs	37	Normal vs. 81-100 yrs	8.08880000000567E-05
Histological subtype	Adenocarcinoma	264	Normal-vs-Adenocarcinoma	4.115900E-03
	Mucinous adenocarcinoma	44	Normal-vs-Mucinous-adenocarcinoma	1.216190E-01
Nodal metastasis status	N0-No regional lymph node metastasis	185	Normal-vs-N0	9.93780000000388E-05
	N1- Metastases in 1 to 3 axillary lymph nodes	74	Normal-vs-N1	6.166200E-01
	N2-Metastases in 4 to 9 axillary lymph nodes	50	Normal-vs-N2	4.580600E-01
TP53 mutation status	TP53 mutant	174	Normal-vs-TP53-Mutant	4.651500E-02
	TP53 non-mutant	136	Normal-vs-TP53-NonMutant	1.052560E-01

Suppl.Table 4- Coexpression and Correlation of the top 25 gene associated with COL11A1 gene in colorectal cancer using cBioPortal. The p-value is derived from two-sided t-test ad the q-value derived from Benjamini-Hochberg FDR correction procedure.

Ranking	Correlated Gene	Cytoband	Spearman's Correlation	p-Value	q-Value
1	THBS2	6q27	0.922	4.83e-217	9.66e-213
2	COL10A1	6q22.1	0.913	1.18e-205	1.18e-201
3	COL5A2	2q32.2	0.909	9.10e-201	6.07e-197
4	COL1A2	7q21.3	0.903	1.61e-193	8.07e-190
5	SULF1	8q13.2-q13.3	0.901	2.06e-191	8.26e-188
6	COL3A1	2q32.2	0.900	3.11e-190	1.04e-186
7	NTM	11q25	0.898	6.84e-188	1.96e-184
8	ADAM12	10q26.2	0.897	3.88e-187	9.70e-184
9	CTHRC1	8q22.3	0.891	2.60e-181	5.79e-178
10	VCAN	5q14.2-q14.3	0.881	9.67e-172	1.94e-168
11	FBN1	15q21.1	0.878	9.58e-169	1.74e-165
12	POSTN	13q13.3	0.874	9.20e-166	1.53e-162
13	ANTXR1	2p13.3	0.871	6.93e-163	1.07e-159
14	RAB31	18p11.22	0.870	9.29e-163	1.33e-159
15	SPOCK1	5q31.2	0.870	4.94e-162	6.60e-159
16	SPARC	5q33.1	0.867	4.10e-160	5.13e-157
17	ST6GALNAC5	1p31.1	0.864	4.70e-158	5.53e-155
18	FNDC1	6q25.3	0.864	1.43e-157	1.59e-154
19	FAP	2q24.2	0.863	3.58e-157	3.77e-154
20	COL5A1	9q34.3	0.863	6.83e-157	6.84e-154
21	PLPP4	10q26.12	0.862	2.40e-156	2.29e-153
22	NOX4	11q14.3	0.861	1.67e-155	1.52e-152
23	COL1A1	17q21.33	0.858	6.15e-153	5.36e-150
24	CCDC8	19q13.32	0.857	2.36e-152	1.92e-149
25	ITGA11	15q23	0.857	2.40e-152	1.92e-149

Suppl.Table 5.- Gene Network Enrichment of Top 25 correlated genes of COL11A1 gene using NetworkAnalyst server for GO: biological process, GO: molecular function, Reactome pathways and KEGG pathways network.

**Pathways name**	**Hits**	**P-value**	**AdjP**
**GO:Biological Process**			
Extracellular structure organization	10/242	6.76e-12	5.54e-9
Skeletal system development	9/459	6.47e-8	2.65e-5
Heart development	7/487	1.92e-5	0.00524
Cell migration	9/1050	6.19e-5	0.00993
Cell_substrate adhesion	5/241	6.45e-5	0.00993
System development	19/4950	7.27e-5	0.00993
Neuron development	8/945	0.00019	0.0181
Cell development	11/1840	1.93e-4	0.0181
Anatomical structure development	20/5830	1.99e-4	0.0181
Epidermis development	5/319	0.00024	0.0197
Tissue morphogenesis	6/566	0.00044	0.0284
Response to endogenous stimulus	9/1360	4.46e-4	0.0284
Tissue development	10/1680	4.51e-4	0.0284
Neurogenesis	9/1390	5.16e-4	0.0302
Organ development	14/3290	6.36e-4	0.0314
Multicellular organismal development	19/5720	0.00065	0.0314
Neuron differentiation	8/1190	8.85e-4	0.04
Vasculature development	6/652	9.28e-4	0.04
Proteoglycan metabolic process	3/117	0.00121	0.0497
Anatomical structure morphogenesis	12/2820	0.00203	0.0693
Anatomical structure formation involved in morphogenesis	10/2090	0.00247	0.081
Cell morphogenesis involved in differentiation	6/827	0.00313	0.0951
Negative regulation of angiogenesis	2/56	0.00462	0.122
Transmembrane receptor protein serine/threonine kinase signaling pathway	4/388	0.00499	0.128
Transforming growth factor beta receptor signaling pathway	3/221	0.0073	0.176
Wound healing	5/700	0.00776	0.182
Response to external stimulus	7/1510	0.0157	0.331
Blood coagulation	4/564	0.018	0.356
tissue remodeling	2/126	0.0218	0.407
Response to wounding	6/1310	0.0276	0.482
Cell_matrix adhesion	2/159	0.0336	0.54
Regulation of anatomical structure morphogenesis	4/702	0.0365	0.576
Regulation of angiogenesis	2/169	0.0375	0.581
Morphogenesis of an epithelium	3/440	0.0446	0.678
Regulation of cell migration	3/456	0.0488	0.727

**GO: Molecular Function**			
Extracellular matrix structural constituent	7/80	3.85e-11	1.5e-8
Growth factor binding	4/125	4.92e-5	0.00713
Collagen binding	3/47	6.18e-5	0.00713
Structural molecule activity	7/666	7.35e-5	0.00713
Cation binding	16/4160	1.27e-4	0.00984
SMAD binding	3/68	1.87e-4	0.0107
Glycosaminoglycan binding	4/178	1.93e-4	0.0107
Ion binding	19/6140	2.72e-4	0.0132
Calcium ion binding	6/673	6.46e-4	0.0279
Heparin binding	3/130	0.00125	0.0484
Integrin binding	2/86	0.00884	0.312
Metalloendopeptidase activity	2/123	0.0175	0.565
Protein binding, bridging	2/135	0.0208	0.621
Arylsulfatase activity	1/15	0.0246	0.639
Binding, bridging	2/148	0.0247	0.639
Sulfuric ester hydrolase activity	1/26	0.0422	1
Sialyltransferase activity	1/27	0.0438	1
**Reactome Pathway**			
Assembly of collagen fibrils and other multimeric structures	7/54	4.97e-12	6.41e-9
Degradation of collagen	7/61	1.22e-11	6.41e-9
Collagen biosynthesis and modifying enzymes	7/62	1.37e-11	6.41e-9
Degradation of the extracellular matrix	7/77	6.61e-11	2.32e-8
Collagen formation	7/85	1.35e-10	3.78e-8
Extracellular matrix organization	8/157	2.08e-10	4.87e-8
Integrin cell surface interactions	4/85	2.22e-5	0.00445
NCAM1 interactions	3/39	6.42e-5	0.0112
Crosslinking of collagen fibrils	2/11	2.22e-4	0.0346
Platelet Adhesion to exposed collagen	2/12	2.66e-4	0.0373
NCAM signaling for neurite out-growth	3/65	2.97e-4	0.0378
Anchoring fibril formation	2/15	4.22e-4	0.0493
Signaling by PDGF	4/189	4.98e-4	0.0537
GPVI-mediated activation cascade	2/33	0.00208	0.208
Signal Transduction	9/1690	0.00232	0.217
Platelet activation, signaling and aggregation	3/220	0.00981	0.86
Cell surface interactions at the vascular wall	2/99	0.0176	1
Dermatan sulfate biosynthesis	1/10	0.0208	1
Axon guidance	3/292	0.021	1
CS/DS degradation	1/14	0.029	1
Chondroitin sulfate biosynthesis	1/23	0.0472	1
**KEGG Pathway**			

Protein digestion and absorption	7/90	1.36e-10	4.32e-8
ECM-receptor interaction	4/82	1.46e-5	0.00233
AGE-RAGE signaling pathway in diabetic complications	4/100	3.22e-5	0.00341
Focal adhesion	4/199	4.64e-4	0.0369
Amoebiasis	3/96	7.56e-4	0.0481
Platelet activation	3/124	0.00159	0.0842
PI3K-Akt signaling pathway	4/354	0.00394	0.179
Glycosphingolipid biosynthesis - ganglio series	1/15	0.0287	1
Malaria	1/49	0.091	1
Glycerolipid metabolism	1/61	0.112	1

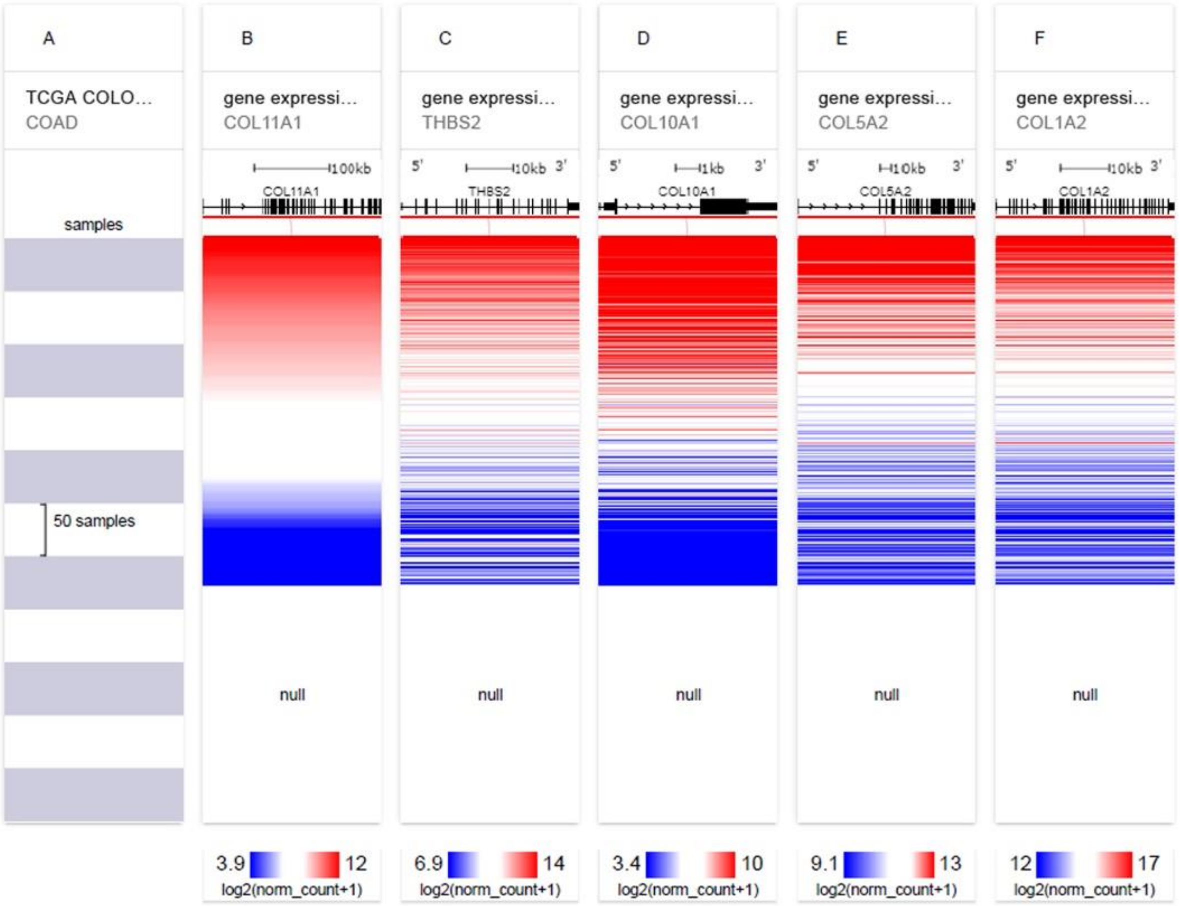
A
B
C
D
E
F
TCGA COLO...
COAD
gene expressi...
COL11A1
gene expressi...
THBS2
gene expressi...
COL10A1
gene expressi...
COL5A2
gene expressi...
COL1A2
samples
50 samples
null
3.9
12
6.9
14
3.4
10
9.1
13
12
17
log2(norm_count+1)

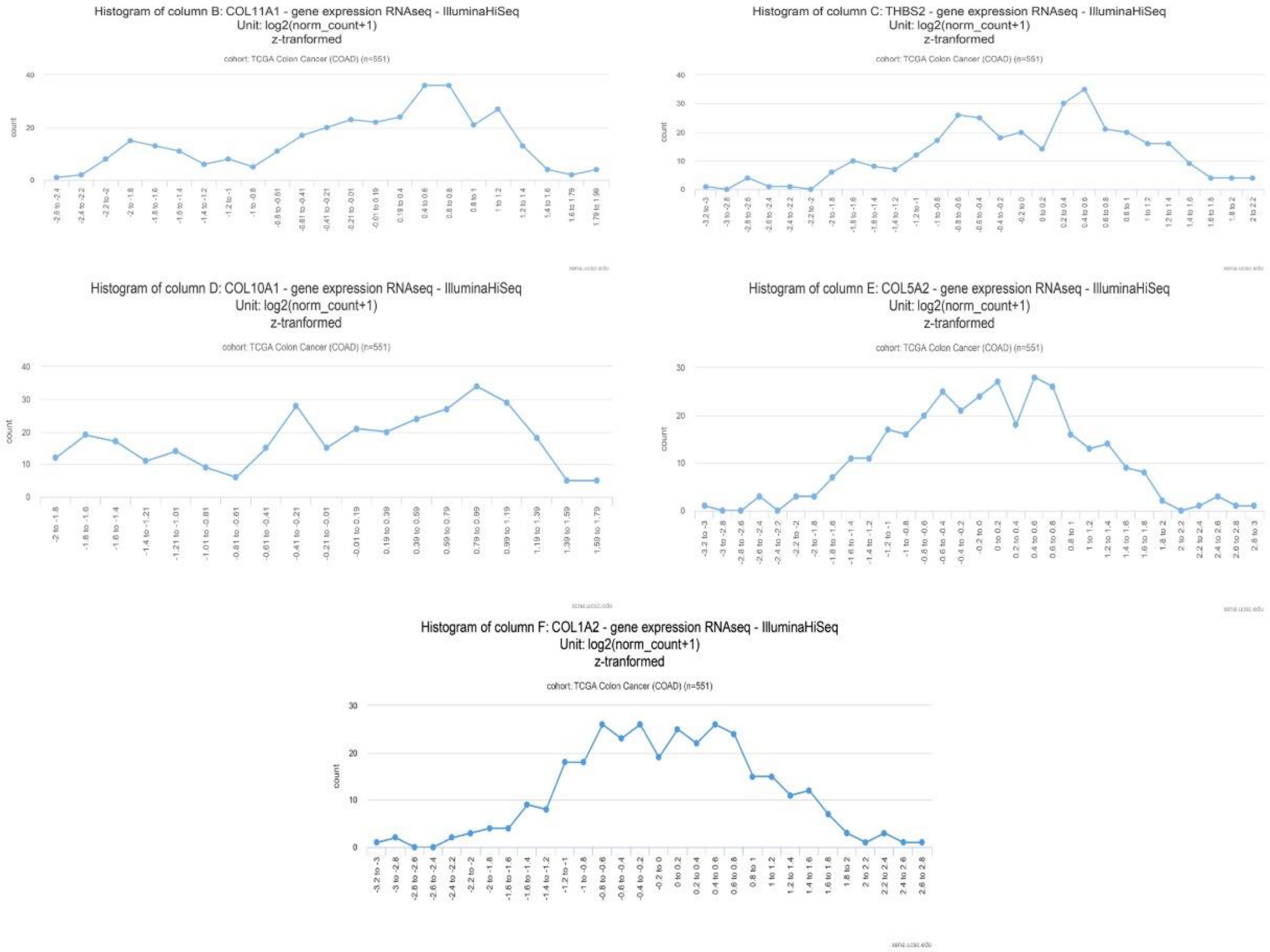
Histogram of column B: COL11A1 - gene expression RNAseq - IlluminaHiSeq
Unit: log2(norm_count+1)
z-tranformed
cohort: TCGA Colon Cancer (COAD) (n=551)
count
Histogram of column C: THBS2 - gene expression RNAseq - IlluminaHiSeq
Unit: log2(norm_count+1)
z-tranformed
cohort: TCGA Colon Cancer (COAD) (n=551)
count
Histogram of column D: COL10A1 - gene expression RNAseq - IlluminaHiSeq
Unit: log2(norm_count+1)
z-tranformed
cohort: TCGA Colon Cancer (COAD) (n=551)
count
Histogram of column E: COL5A2 - gene expression RNAseq - IlluminaHiSeq
Unit: log2(norm_count+1)
z-tranformed
cohort: TCGA Colon Cancer (COAD) (n=551)
count
Histogram of column F: COL1A2 - gene expression RNAseq - IlluminaHiSeq
Unit: log2(norm_count+1)
z-tranformed
cohort: TCGA Colon Cancer (COAD) (n=551)
count

Suppl. Figure 1. Heatmap and histogram for the gene expression of COL11A1, THBS2, COL10A1, COL5A2, and COL1A2 in the TCGA COAD dataset using UCSC XENA. The red and blue color represents the high and low expression in the heatmap. The histogram represents the gene expression with z-score transformation.

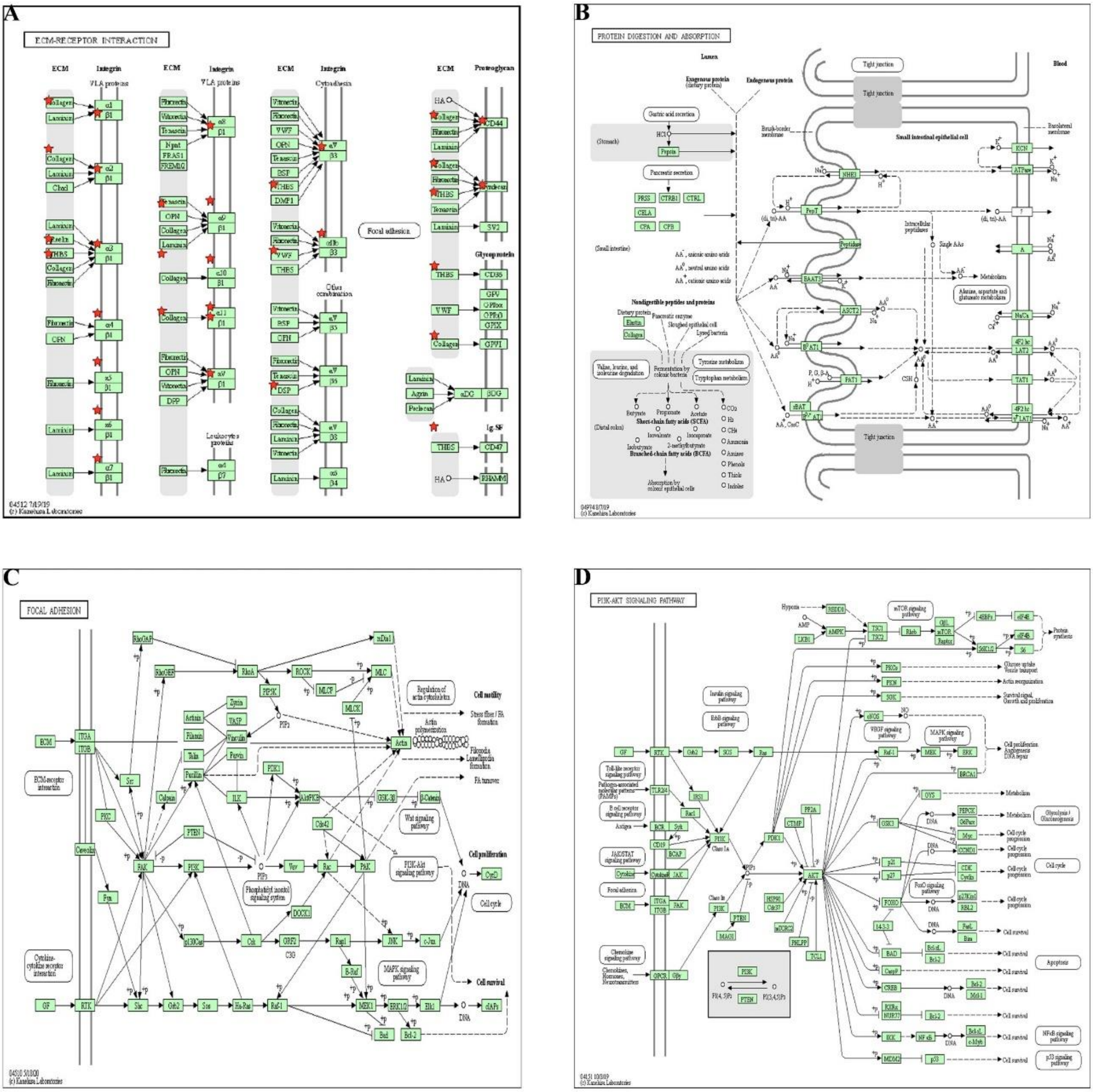

Suppl. Figure 2. Intervention of top 25 correlated genes on the KEGG pathways using DAVID. **A.** ECM-receptor interaction, **B.** Protein digestion and absorption, **C.** Focal-adhesion, **D.** PI3K-Akt signaling pathway.
